# Assessing the Efficacy of VLP-Based Vaccine against Epstein-Barr Virus Using a Rabbit Model

**DOI:** 10.3390/vaccines11030540

**Published:** 2023-02-24

**Authors:** Narendran Reguraman, Asma Hassani, Pretty S. Philip, Dagmar Pich, Wolfgang Hammerschmidt, Gulfaraz Khan

**Affiliations:** 1 Department of Microbiology and Immunology, College of Medicine and Health Sciences, United Arab Emirates University, Al Ain P.O. Box 15551, United Arab Emirates; 2 Research Unit Gene Vectors, Helmholtz Zentrum München, German Research Center for Environmental Health, 25, D-81377 Munich, Germany; 3 Zayed Center for Health Sciences, United Arab Emirates University, Al Ain P.O. Box 15551, United Arab Emirates

**Keywords:** EBV, vaccine, VLP, rabbits, booster dose, immune response

## Abstract

Epstein–Barr virus (EBV) is etiologically associated with a number of malignant and non-malignant conditions. Thus, a prophylactic vaccine against this virus could help to reduce the burden of many EBV-associated diseases. Previously, we reported that an EBV virus-like particle (VLP) vaccine was highly immunogenic and produced a strong humoral response in mice. However, since EBV does not infect mice, the efficacy of the VLP in preventing EBV infection could not be addressed. Here we examined, for the first time, the efficacy of the EBV-VLP vaccine using a novel rabbit model of EBV infection. Animals vaccinated with two doses of VLP elicited higher antibody responses to total EBV antigens compared to animals receiving one dose. Vaccinated animals also elicited both IgM and IgG to EBV-specific antigens, VCA and EBNA1. Analysis of peripheral blood and spleen for EBV copy number indicated that the viral load in both of these compartments was lower in animals receiving a 2-dose vaccine. However, the VLP vaccine was ineffective in preventing EBV infection. With several other EBV vaccine candidates currently at various stages of development and testing, we believe that the rabbit model of EBV infection could be a great platform for evaluating potential candidates.

## 1. Introduction

Epstein–Barr virus (EBV), also known as human herpesvirus 4, is an oncogenic virus infecting about 90% of the human population. Primary EBV infection typically occurs early in childhood and establishes lifelong latency in memory B cells without triggering any symptomatic disease [1,2]. However, this harmless image of EBV infection contradicts its factual pathological potential. The subtle balance of virus-host interaction can be disturbed in several ways, which can lead to a range of diseases, including infectious mononucleosis [3,4], multiple sclerosis [5,6,7,8], post-transplant lymphoproliferative disease [9,10], and a number of malignancies of B lymphocyte and epithelial cell origin [11,12]. It is estimated that more than 250,000 cases of cancer are annually attributed to EBV [13,14]. In immunocompromised individuals, EBV is an important contributing factor to the overall morbidity and mortality. For example, patients who acquire primary EBV infection following transplantation are at increased risk of developing EBV-associated malignancies. Thus, a prophylactic vaccine could have a major impact on reducing the burden of not only EBV-associated malignancies, but also EBV-associated non-malignant conditions such as infectious mononucleosis and multiple sclerosis [15,16,17]. 

Over the past five decades, various vaccine candidates have been tried and tested. However, of all the vaccines that have been rigorously tested, none have so far made it to the market. These vaccine candidates are either not immunogenic enough to raise neutralizing antibodies or cannot stop the virus from infecting the host [18,19,20,21,22]. Following the success of the virus-like particle (VLP) vaccine in preventing HPV infection [23], the possibility of a VLP vaccine against EBV was also explored. Since VLP vaccines are free of the viral genome, they are considered relatively safe. Moreover, they possess key features of the virus that can elicit an immune response [24]. In 2011, a new generation of packaging cell line (293-VII+) for EBV vectors was reported [25]. In this cell line, viral genes, such as LMP1, EBNA2, EBNA3A, 3B, 3C, and BZLF1, were deleted or functionally inactivated to avoid either virus production or B cell transformation [25]. The EBV VLPs produced in this cell line, however, were believed to contain sufficient quantities of EBNA1 and LMP2. These VLPs were found to be highly immunogenic, eliciting humoral as well as strong CD8+ and CD4+ T cell responses [25]. 

Although EBV VLPs were shown to induce a robust immune response, both in vitro and in the murine model, there were some limitations. Murine B cells are not susceptible to EBV infection, and their response varies compared to humans. As suggested in our previous study, further research on non-human primates was needed to determine the actual potential of this VLP vaccine in preventing EBV infection [25]. However, due to the limited availability, high cost, and endangered status, the use of primates is much more challenging. Hence, alternative models for EBV infection are highly preferred. Phylogenetically, rabbits are the next closest relative to primates. Moreover, compared to mice and rats, rabbits share more similar physiological characteristics with humans [26]. Recently, we reported that healthy rabbits are susceptible to EBV infection, and the infection resembles what is observed in humans [27]. Here we use the rabbit model to address the efficacy of the EBV VLPs in response to primary EBV infection. 

## 2. Materials and Methods

### 2.1. Animals 

Healthy New Zealand white rabbits aged approximately 4–6 weeks, weighing 500–800 g, were purchased from a local vendor and housed in the animal facility of the College of Medicine and Health Sciences, UAE University. In our hands, only young animals were reproducibly susceptible to EBV infection [27]. All animals were tagged, weighed, and kept for approximately 2 weeks to acclimatize to the new environment before performing any experiments. All experiments were conducted in accordance with guidelines provided by the Institutional Animal Research Ethics Committee of UAE University and approved by the Institutional Review Board (approval #A15-15: ERA 2018-5718). 

### 2.2. Experimental Design

Animals were randomly divided into three groups, as follows:

Vaccinated and Infected Group (VIG) (n = 7). These animals received the EBV VLP vaccine, either one dose (n = 3) or two doses (n = 4) 1-week apart. All animals were subsequently challenged with EBV infection, either 2-weeks (animals receiving 1 dose) or 4 weeks (animals receiving 2 doses) after the last dose of the vaccine and sacrificed 2 weeks post-infection. 

Non-vaccinated and Infected Group (NVIG) (n = 3). Based on our previous studies, healthy rabbits are susceptible to EBV infection [27]. Hence, we limited the number of animals to just 3 for this group. These animals received physiological saline (PBS) instead of the vaccine. However, all three animals received the same inoculum of EBV as the vaccinated group.

Non-vaccinated and Non-Infected Group (NVNIG) (n = 3). These animals were our “negative control.” They received PBS in place of VLP or EBV.

### 2.3. Generation, Purification, and Quantification of VLPs

The generation of VLPs and the construction of packaging cell lines (293-VII+) has been described in our previous study [25]. Briefly, the entire genome of EBV was cloned into a BACmid, also known as Maxi EBV [28]. This VLP-Maxi EBV DNA was stably transfected into HEK 293 cells. Single-cell clones were isolated and selected using puromycin. A single clone was identified as the best VLP producer. In this cell clone, VLP production was induced by transient transfection of plasmid DNA expressing BZLF1 to switch from viral latency to the lytic, productive phase of EBV [28]. The culture medium was exchanged 12 h after transfection to plain RPMI1640 medium supplemented with 4 g/L glucose. VLPs released into the supernatant were harvested after 4 days.

EBV VLPs from the culture supernatant were collected by ultracentrifugation and purified using density gradient ultracentrifugation using Optiprep as described [29]. Gradient fractions containing the VLPs were concentrated again by ultracentrifugation and resuspended in PBS. Independent analysis using mass spectrometry indicated that the VLPs contained approximately 50 viral proteins in different concentrations mimicking the composition of B95-8 virus stocks [30].

VLPs were quantified using the Elijah cell binding assay and the infectious GFP encoding 2089 EBV strain as reference [31]. The 2089 virus reference was used to express VLPs concentrations as equivalents to infectious B95-8 EBV stocks. The resulting VLP pellets were washed, resuspended in PBS, quantitated, and stored at −80 °C until required. In the process of creating an efficient non-transforming, virus-free packaging cell line 293-VII, Hettich et al. deleted or functionally inactivated 6 EBV proteins, LMP1, EBNA2, 3A, 3B, 3C, and BZLF1. This was further confirmed by comparative PCR analysis of selected gene loci in the packaging cell line 293-VII. [32]. However, these VLPs contained an adequate amount of EBNA1 and LMP2 to induce EBV-specific immune response.

### 2.4. Immunization of Rabbits with VLPs

Animals were given intramuscular injections of 7 × 10^7^ VLPs in 500 μL sterile PBS. For animals receiving 2 doses, the same amount of VLPs were given one week after the first dose. Blood was collected into heparin tubes from each animal before starting the experiment and on the day of sacrifice. Peripheral blood mononuclear cells (PBMCs) and plasma were isolated from whole blood using Histopaque 1083 (Sigma, Saint Louis, MO, USA). The PBMCs and plasma were then stored in liquid N_2_ and −40 °C, respectively, till further analysis. 

### 2.5. Preparation of EBV Inoculum

EBV producer cell line (B95-8 cells) was cultured in RPMI media (GIBCO, Waltham, MA, USA), supplemented with 10% FBS (GIBCO, Waltham, MA, USA), 1% antibiotic and antimycotic solution (Santa Cruz, CA, USA), 50 µg/mL gentamycin (Hyclone, Logan, GA, USA), and 1× glutamine (GIBCO, Waltham, MA, USA) at 37 °C, and 5% CO_2_, as previously described [27]. Cells were cultured until they attained high density (6.5 × 10^6^ cells/mL). Culture supernatants were centrifuged to remove cells/cell debris and then filtered using a 0.2 μm filter. Fresh-filtered virus preparations were used for inoculating rabbits. EBV copy number in the inoculum was estimated by quantitative real-time PCR (qPCR) [27]. 

### 2.6. Estimation of EBV Copy Number and Inoculation

Animals were injected intravenously with 1000 µL of fresh culture supernatant filtrate containing the virus in the marginal ear vein (500 µL in each ear). Each animal was infected with approximately 5 × 10^6^ EBV copies. EBV copy number in the initial inoculum and in the rabbit PBMCs/spleen tissues was estimated using qPCR targeting EBV BamH1W region. The Namalwa cell line was used as the DNA standard, as previously described [27]. Namalwa is a Burkitt’s lymphoma cell line that has a known number of copies of EBV DNA in each cell (2 integrated copies). Briefly, a standard curve was created utilizing gDNA from Namalwa cells to determine EBV copy number in test samples [33]. Five 10-fold serial dilutions of Namalwa DNA (100, 10, 1, 0.1, and 0.01 ng/L) were used to create the standard curve. The logarithm of Namalwa DNA quantities was plotted against the mean of the cycle threshold (Ct). Each qPCR reaction contained 50 ng of template DNA in a total reaction volume of 20 μL with TaqMan Universal Master Mix and TaqMan probe (Table S1). All samples were tested in duplicates or triplicates in a 40-cycle reaction using Applied Biosystems (QuantStudio-7) real-time PCR machine, and the mean copy number was calculated for each sample [27]. 

### 2.7. Detection of EBV Using EBER In Situ Hybridization 

The cellular localization of EBV in the autopsied tissues was determined using the highly sensitive technique of EBER-in situ hybridization (EBER-ISH), as previously described [34,35]. Briefly, EBER-ISH was performed on 5 μm-thick formalin-fixed, paraffin-embedded (FFPE) tissue sections using a mixture of digoxigenin-labeled probes complementary to EBER-1 and EBER-2 [34]. These two non-protein-coding small RNAs are expressed at millions of copies in EBV-infected cells. Thus, targeting EBERs has become the gold standard in the detection of EBV in histological tissues. Following the blocking of endogenous peroxidase activity, sections were briefly digested with 0.1 mg/mL of proteinase K (Sigma, Saint Louis, MO, USA) and then hybridized overnight with the labeled EBER probes. Hybridized probes were subsequently detected using mouse anti-digoxin monoclonal antibody (D1-22, Sigma, Saint Louis, MO, USA) at a dilution of 1/2500 and the Ultra-Sensitive ABC-Peroxidase Staining kit (Thermo Fisher Scientific, Waltham, MA, USA). Diaminobenzidine tetrahydrochloride (DAB) was used as the chromogen. With each batch of tissue sections, a positive control tissue (EBV-infected B95-8 cells or EBV-lymphoblastoid cell lines) and a negative control probe (using digoxigenin-labeled non-complimentary EBER probes) were included.

### 2.8. Detection of EBV Protein/Gene Expression Using Immunohistochemistry and qPCR

Immunohistochemistry (IHC) was performed on 5 μm sections of FFPE rabbit spleens [27]. Sections were deparaffinized, dehydrated, and blocked for endogenous peroxidase. The slides were then rehydrated and kept in boiling citrate buffer for antigen retrieval, followed by cooling to room temperature on a benchtop. Tissues were then blocked using 5% BSA for 1 h and then incubated overnight in primary monoclonal antibodies at pre-optimized dilutions [27]. The following EBV proteins were detected by IHC: LMP1 (CS1-4 at 1:1000, Abcam UK), EBNA1 (D810H at 1:20, Thermo Scientific, Waltham, MA, USA), and BZLF1 (BZ1 at 1:200, Santa Cruz, CA, USA). Following incubation with the primary antibodies, sections were washed and incubated with conjugated anti-mouse secondary antibody (Ultra-Sensitive ABC-Peroxidase Staining kit, Thermo Fisher Scientific, Waltham, MA, USA), and the signal was developed using a chromogenic DAB detection system. 

Quantitative PCR was used to determine EBV gene expression in the spleen of rabbits using Power SYBR^®^ Green PCR Master Mix (Applied Biosystems). All the samples were either tested in duplicates or triplicates and independently repeated 3 times in a 40-cycle reaction. References included rabbit specific GAPDH (housekeeping gene) and non-infected PBMC samples (experimental controls). The sequences of the primers used are listed in Table S2.

### 2.9. Detection of Anti-EBV Antibodies in Plasma

A modified Enzyme-linked immunosorbent assay (ELISA) was used to determine EBV-specific antibodies in rabbit plasma [27]. Briefly, total proteins were first isolated from the EBV-producing cell line, B95-8 cells. Each well of a 96-well plate was then coated with 100 ng of the extracted protein in PBS and incubated overnight at 4 °C. Additionally, commercially available ELISA kits from Trinity biotech (Captia IgM and IgG) were used to detect EBV-specific antibodies against EBNA1 and VCA. Plasma from rabbits, at various dilutions, was added to the wells. The bound antibodies were then detected using their respective HRP-conjugated secondary antibodies and developed using TMB substrate. Upon developing the optimal color intensity, the reaction was stopped using 0.1N H_2_SO_4_, and the OD was measured at 450 nm. The ELISA results were normalized with pre-treatment control samples and plotted against their respective ODs.

### 2.10. Statistical Analysis

Statistical analyses were performed using GraphPad Prism Version 9.1.2. Results were expressed as mean ± standard error of the mean (SEM). Statistical significance was determined using one-way ANOVA and two-way ANOVA (viral load) followed by the Mann–Whitney test. Differences were considered significant if *p* < 0.05.

## 3. Results

### 3.1. Clinical Features

Following VLP vaccination and/or EBV infection, all animals were regularly monitored for any signs of illness, including diarrhea and weight loss. All the animals remained healthy, and no signs of illness were observed. Furthermore, at autopsy, no macroscopic abnormality was observed in any of the organs examined. The average size of the spleen in the vaccinated and infected animals was similar to that of the non-vaccinated, non-infected animals (3–4 cm).

### 3.2. Animals Vaccinated with Two Doses of VLP Triggered Higher Antibody Response to Total EBV Antigens Compared to Animals Receiving One Dose of VLP

**Figure 1:**
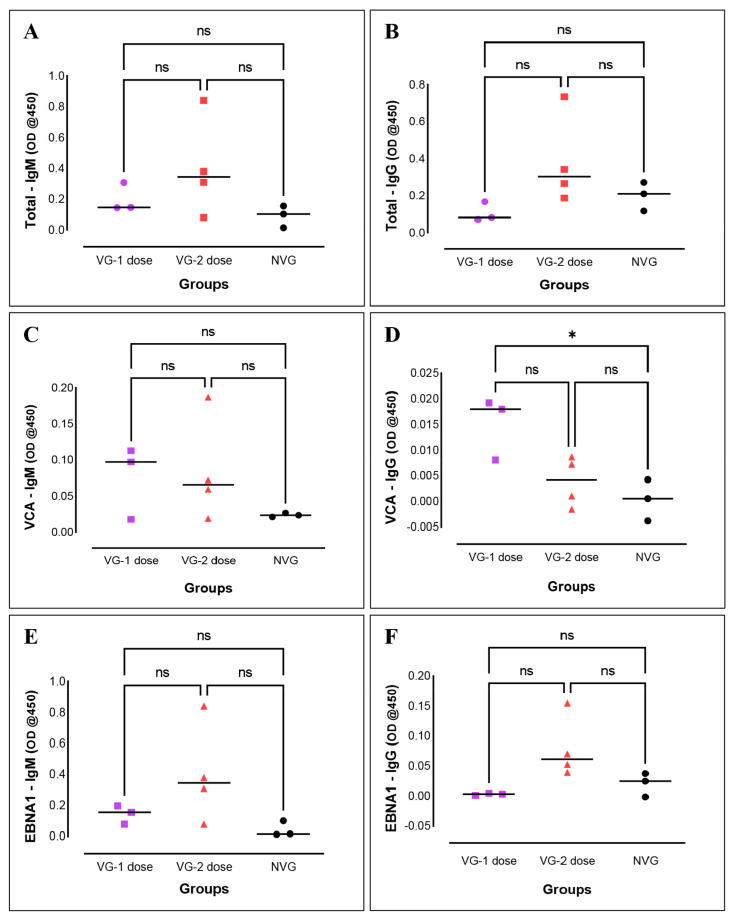
Antibody response in rabbits to EBV proteins. IgM and IgG immune response against total EBV proteins in rabbits immunized with VIG 1-dose, VIG 2-dose, and NVIG (**A**,**B**) and EBV specific IgM and IgG immune responses against VCA (**C**,**D**) and EBNA1 (**E**,**F**). All the data were normalized with the NVNIG controls and plotted against their respective ODs. Antibody titers are presented with mean ± SEM, and comparisons were made using paired *t*-tests. ns: *p* > 0.05, * *p* ≤ 0.05.

To determine whether any immune response was elicited against the VLP vaccine, total antibody levels were measured in the plasma of the vaccinated and infected group (VIG), non-vaccinated and infected group (NVIG), and non-vaccinated and non-infected group (NVNIG). We used the total protein lysate extracted from the B95-8 to coat the ELISA plates [27]. Antibody titers in vaccinated and non-vaccinated EBV-infected animals (VIG and NVIG) were normalized to NVNIG controls. All VIG and NVIG animals triggered IgM and IgG responses to EBV proteins. The levels of titers were higher in animals receiving two doses of the VLP vaccine compared to those receiving one dose (Figure 1A,B). However, the differences were not statistically significant. 

### 3.3. VLP Vaccinated Animals Triggered Both IgM and IgG to EBV-Specific Proteins

To determine whether immunized rabbits elicited any immune response against specific EBV antigens, we performed ELISA for VCA and EBNA1. We found that rabbits immunized with either 1 dose or 2 doses of VLPs triggered both IgM and IgG responses to VCA and EBNA1. For VCA, both IgM and IgG titers were higher in 1-dose VLP animals compared to 2-dose VLP animals (Figure 1C,D). By contrast, for EBNA1, both IgM and IgG titers were higher in the animals which received two doses. Once again, the differences in IgM and IgG levels in animals that received 1-dose or 2-dose VLPs were not significant, either for VCA or for EBNA1. Indeed, the only significant difference observed was for anti-VCA IgG between 1-dose vaccinated animals and NVIG (Figure 1D).

### 3.4. Determination of EBV Viral Load and Gene Expression

**Figure 2:**
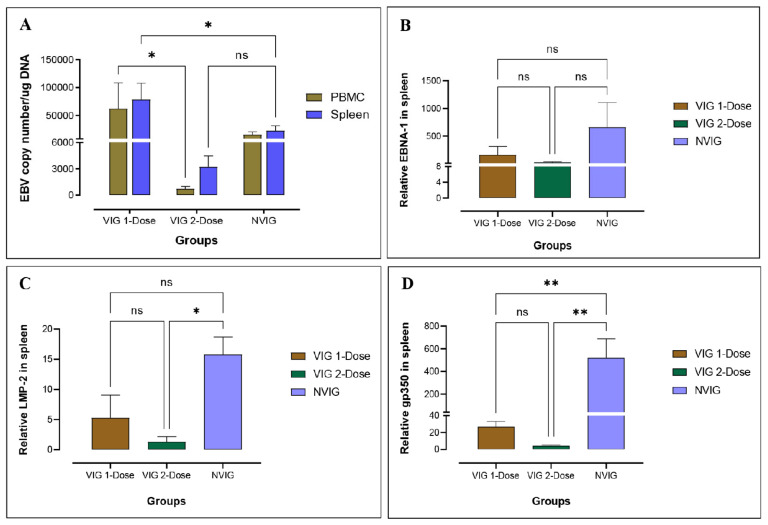
Determination of viral load and EBV gene expression in rabbits. Comparison of viral load by qPCR in the spleen and PBMCs of VIG 1-dose and VIG 2-dose compared to NVIG (**A**). EBV gene expression in VIG 1-dose and VIG 2-dose compared to NVIG for EBNA1 (**B**), LMP-2 (**C**), and gp350 (**D**) in the rabbit spleen. All the data here are normalized with NVNIG. ns: *p* > 0.05, * *p* ≤ 0.05, ** *p* ≤ 0.01.

To determine the viral load of EBV in PBMCs and spleen, qPCR was performed using the Namalwa cell line as the standard [27]. EBV was detected in the spleen and PBMCs of all animals in the VIG 1-dose, VIG 2-dose, and NVIG controls (Figure 2A). However, in 2-dose vaccinated animals, the viral load was more than 10-fold lower than in the 1-dose vaccinated animals.

Analysis of EBV gene expression by RT-PCR indicated the presence of several viral-associated genes in the spleen. EBNA1, LMP2, and gp350 genes were expressed in the spleen of all three groups of animals; VIG 1-dose, VIG 2-dose, as well as NVIG controls (Figure 2B–D). Notably, the non-vaccinated animals (NVIG) expressed higher levels of these genes compared to the vaccinated animals. Thus, although the VLP vaccine reduced the viral load and viral gene expression, it did not prevent infection.

### 3.5. Vaccinated Rabbits Were Not Protected against EBV Infection

**Figure 3:**
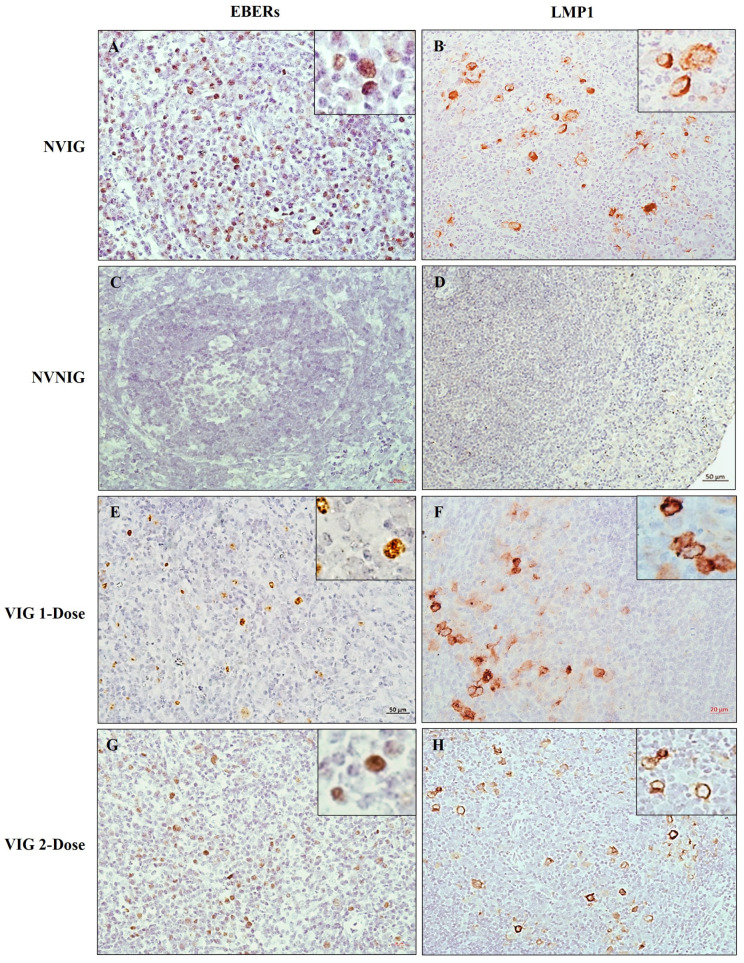
Detection of EBV and its gene expression in rabbit spleen. EBER-in situ hybridization was used to detect EBERs, and immunohistochemistry was used to detect the expression of LMP-1. Rabbits in the non-vaccinated, infected group (NVIG) were clearly positive for both EBERs (**A**) and for LMP1 expression (**B**), whilst the non-vaccinated, non-infected control (NVNIG) animals were negative for both (**C**,**D**). Animals vaccinated with either 1-dose of the VLP vaccine (**E**,**F**) or 2-doses (**G**,**H**) and subsequently challenged with EBV were not protected from infection. The spleens in animals from both groups were positive for EBERs and LMP1.

EBER in-situ hybridization (EBER-ISH) and LMP1 immunohistochemistry were performed on the spleen of all animals, VIG, NVIG, and NVNIG, to assess the extent of EBV infection. As expected, NVIG animals were clearly susceptible to EBV infection (Figure 3A,B), whilst NVNIG animals were clearly negative (Figure 3C,D). Disappointingly, vaccinated animals (VIG 1-dose or 2-dose) were not protected from EBV infection (Figure 3E–H). Interestingly, we observed occasional BZLF1positive cells in the spleen of NVIG animals but not in the vaccinated animals (Figure S1). Irrespective of the dose (1-dose or 2-dose), all VIG animals remained healthy and active during the entire course of the experiment. Thus, the VLP vaccine was safe but not effective in preventing EBV infection. 

## 4. Discussion

EBV was discovered well over half a century ago from a case of Burkitt’s lymphoma [36]. The central role of this oncogenic virus in the pathogenesis of various malignancies and the importance of developing a prophylactic vaccine was realized very early on [37]. Over the past five decades, this list of malignant and non-malignant conditions attributed to EBV has increased considerably [38]. However, developing an effective vaccine has remained frustratingly difficult. 

Numerous vaccine candidates have been investigated over the years with varying success [16,39]. For example, the vaccinia virus constructs expressing EBV gp220-340 were tested in the phase I trial, and although it presented with good efficacy, no further work was continued, possibly due to the adverse effects of using live vaccinia virus [40]. A considerable number of other studies have focused on EBV gp350. This glycoprotein is expressed in abundance on the viral envelope, and it mediates the entry of the virus into B-cells by binding to CD21. Several subunit vaccines containing soluble gp350 reached phase 1/2 clinical trials with and without adjuvants [18,19,21,40,41]. However, in these trials, either the vaccine did not completely prevent infection, or it did not raise sufficient levels of neutralizing antibodies. Epitope-based CD8+ T cell vaccines using primarily EBV nuclear antigens have also been investigated [20,42,43]. Although these vaccines appear to be useful in preventing PTLD in transplant patients, their use is limited due to their restrictive targeting capacity. Furthermore, studies using monomeric or tetrameric subunits have shown that vaccines with tetrameric gp350 subunits produce higher responses than monomeric subunits [44]. Since EBV gH/gL and gB are involved in EBV fusion to B cells and epithelial cells, recombinant vaccines containing these glycoproteins could prevent EBV infection of epithelial and B cells [22,45]. A recombinant vaccinia virus (MVA-EL) containing two EBV antigens, EBNA1 and LMP-2, was designed to boost T cell immunity against these proteins. Based on the phase 1 clinical trial, Hui et al. reported that the vaccinia-based EBV vaccine could be used as a potential adjuvant treatment for nasopharyngeal carcinoma by combining it with conventional therapies [46]. Other platforms, such as self-assembling nanoparticles, can be used to display totally different viral proteins. Compared to the soluble gp350 vaccine, focusing on the presentation of the CR-2 binding domain of gp350 on nanoparticles induced 10–100 fold higher neutralizing antibodies in mice [47]. Other approaches for targeting EBV have explored the use of EBV-specific T-cells or EBV-specific antibodies [15,48,49]. More recently, it was reported that a VLP vaccine incorporating five EBV glycoproteins, gp350, gB, gp42, gH, and gL, could elicit high titer of antibodies against EBV in rabbits [50]. Although these antibodies were neutralizing in in vitro assays, the ultimate test to examine if this pentavalent EBV-VLP vaccine could prevent EBV infection in rabbits was not performed [50].

Previously, we reported the establishment of a packaging cell line 293-VII, which produced EBV-VLP [25]. This cell line contained the EBV genome with deletions in the genes involved in cell transformation and viral production. The efficacy of EBV-VLPs as a potential vaccine candidate was tested in vitro and in the murine model. The results were very encouraging [25]. EBV VLPs induced a strong immune response, both cellular and humoral, in immunized mice. The VLPs also elicited epitope-specific CD8+ T cell response against BZLF1 and BRLF1 [25]. 

There are other reports on VLPs, where multiple EBV antigens, such as structural and latent proteins were used. These studies suggest that not only can these EBV-VLPs elicit EBV-specific T-cell responses to structural and latent proteins, but they can also provide a higher level of protection in humanized mice [51,52]. One study developed chimeric VLPs incorporating EBV antigens to Newcastle disease virus (NDV) structural proteins [53]. These chimeric NDV-VLPs containing gp350, raised higher immune responses with neutralizing antibodies in mice compared to their monomeric counterpart [53]. Following the development of monomeric gp350 NDV-VLPs, new immunogenic particles with multiple EBV antigens were created using the same principle. This NDV-VLPs polyvalent vaccine was highly immunogenic and raised neutralizing immune responses. T-cell responses specific to EBV proteins were also observed in vaccinated mice [54,55]. Very recently, a bivalent EBV nanoparticle vaccine based on viral glycoproteins that mediate cell entry was reported to elicit neutralizing antibodies in several different animals and inhibited EBV entry into both B cells and epithelial cells [56]. Similarly, a recombinant vesicular stomatitis virus-based EBV vaccine targeting the EBV glycoprotein gB and gHgL was reported to elicit strong neutralizing antibodies [57]. Thus, taken together, these studies highlight the various avenues that are being pursued in the hope of producing a vaccine against EBV that could be used in humans. 

One major obstacle in testing EBV vaccines has been the lack of a natural small animal model of EBV infection. EBV is highly specific, with humans being the primary target. Thus, without the availability of a natural animal model of EBV infection, testing the effectiveness of potential vaccine candidates has been difficult. Recently, several studies, including those from our lab, have reported that rabbits are susceptible to EBV, and the infection recapitulates what has been observed in humans [27,58,59,60,61]. Importantly, it has been reported that in some animals, the virus can persist for life without causing any major pathology [58,59]. In our previous study, we reported that the latent virus could be reactivated after nearly three months, resulting in widespread infection and EBV-driven lymphoproliferation [27]. In this study, we used the rabbit model to evaluate an EBV-VLP-based vaccine from 293-VII, which was previously shown to trigger a robust immune response in in vitro and in murine models [25]. We found that rabbits immunized with two doses of the vaccine triggered IgM and IgG responses against total EBV antigens. Moreover, the vaccine elicited detectable levels of antibodies to EBV antigens EBNA1 and VCA. Indeed, the titers were comparable to the levels seen in animals infected with the virus. Higher viral load in the PBMCs and widespread infection was observed in the spleen of VIG 1-Dose animals, compared with VIG 2-Dose animals. This correlated with higher VCA expression and hence the higher antibody response in 1-Dose animals. However, the VLP vaccine did not protect the animals from subsequent challenges with EBV. The virus was transcriptionally active, and the infected cells expressed a range of EBV latent proteins, including EBNA1, EBNA2, and LMP1.

We suspect that several technical factors could have played a role in the vaccine’s failure to prevent infection. We challenged the animals with EBV after a maximum of 4 weeks following the last dose of the VLP vaccine. It is possible that this duration was insufficient, considering that B cell development in rabbits appears to be different compared to humans [62,63]. Leaving the animals for a longer duration following the second dose would have been preferable. However, in our hands, we found that only young animals are reproducibly susceptible to EBV infection. Hence, we kept the duration between the second dose and EBV challenge to a maximum of 4 weeks to avoid the animals becoming non-susceptible to EBV and hence giving false positive vaccine efficacy. It is also plausible that the VLP vaccine required an adjuvant to trigger an effective response. Adjuvants play various roles in augmenting immunogenicity and improving effectiveness against poorly responding pathogens [64]. A higher response has been reported in patients receiving vaccines adjuvanted with AS04 or aluminum salts compared to non-adjuvanted vaccines [50]. Further, we also observed the EBV copy number to be higher in VIG 1-dose group than in the NVIG group. We believe that the variation observed in the viral load between the VIG 1-dose and NVIG group is most probably due to the small cohort of animals used in this study. Future studies on a larger cohort will help to clarify this variation. Similar to most vaccines, our VLP vaccine was injected intramuscularly. It is possible that other routes of administration may be more effective. Indeed, longer intervals of incubation of adjuvanted VLPs with an extra booster dose (3rd dose), together with the different routes of immunization, have proved effective in a previous report [50]. Thus, future studies should aim to include adjuvants, increase the time duration between the vaccine doses, and consider alternative modes of inoculation, such as IV. Although B-95-8 is a very commonly used source of EBV, it is important to assess if the rabbits are equally susceptible to infection with other strains of the virus. A previous study reported that rabbits were susceptible to P3HR1 stain [65]. However, this strain lacks EBNA2 and is, therefore, not a bona fide representative of wild-type virus infecting humans. We believe further studies, taking these considerations into account, are required to assess if the EBV-VLP vaccine used in this study has any potential for preventing EBV infection.

## 5. Conclusions

Our findings indicate that animals that received two doses of the EBV VLP vaccine elicited a higher response and had lower viral load and gene expression compared to the non-vaccinated animals. Importantly, the VLP vaccine failed to protect rabbits from EBV infection, indicating that further improvements are necessary. Several other vaccine candidates against EBV are currently under investigation, including an mRNA-based vaccine [66], and it is hoped that one of these will eventually make it to the market. In this context, we believe that the rabbit model could be a great platform for evaluating potential vaccine candidates.

## Data Availability

All the relevant data is included in the manuscript.

## Supplementary Materials

The following supporting information can be downloaded at: https://www.mdpi.com/article/10.3390/vaccines11030540/s1, Table S1: The list of primer sequences used to amplify genomic DNA from rabbit tissues. Table S2: List of primers for rabbit cellular transcripts and EBV genes. Figure S1: Detection of EBV lytic gene BZLF1 in rabbit spleen.
