# Supplementary material for: Assessing the Efficacy of VLP-Based Vaccine against Epstein-Barr Virus Using a Rabbit Model
Source: Vaccines (Basel). 2023 Feb 24;11(3):540. doi: 10.3390/vaccines11030540 (PMC10058710; doi:10.3390/vaccines11030540)
Supplement: Supplementary file 1 [file vaccines-11-00540-s001.zip › vaccines-2129704-supplementary.pdf]

SUPPLEMENTARY INFORMATION

Figure S1: Detection of EBV lytic gene BZLF1 in rabbit spleen

Although no BZLF1 expressing cells were observed in the spleen sections of vaccinated rabbits (VIG), we did see occasional BZLF1 positive cell in the spleen of non-vaccinated animals (NVIG) (brown stained cell).

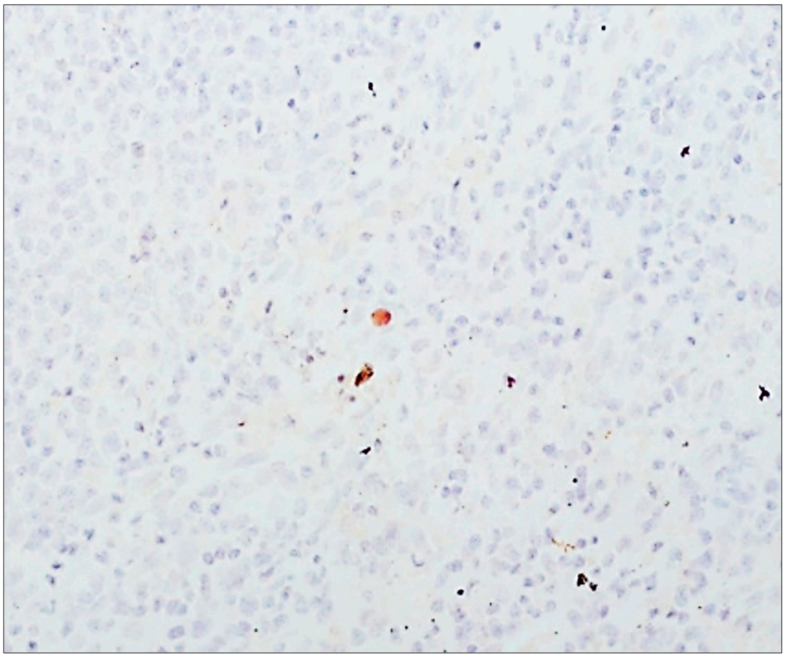

Table S1: The list of primer sequences used to amplify genomic DNA from rabbit tissues.

Gene	Primer sequence
Marmoset β-globin	F: 5′ GAG GTT CTT TGA GTC CTT TGG 3′
	R: 5′ CAT CAC TAA AGG CAC CGA GCA 3'
Rabbit GAPDH	F: 5' GGA GAA AGC TGC TAA 3'
	R: 5' ACG ACC TGG TCC TCG GTG TA3'
EBV BamHI W	F: 5′ GCA GCC GCC CAG TCT CT 3′
	R: 5′ ACA GAC AGT GCA CAG GAG CCT 3′
	Probe: 5′ (6FAM) AAA AGC TGG CGC CCT TGC CTG(TAMRA) 3′

Table S2: List of primers for rabbit cellular transcripts and EBV genes.

Gene	Primer sequence
Rabbit GAPDH	F: 5' TGACGACATCAAGAAGGTGGTG 3'
	R: 5' GAAGGTGGAGGAGTGGGTGTC 3'
gp350	F: 5' AGAATCTGGGCTGGGACGTT 3'
	R: 5' ACATGGAGCCCGGACAAGT 3'
EBNA1	F: 5' GGTCGTGGACGTGGAGAAAA 3'
	R: 5' GGTGGAGACCCGGATGATG 3'
LMP2	F: 5' ATGACTCATCTCAACACATA 3'
	R: 5' CATGTTAAGCAAATTGCAAA 3'
